# Publisher Correction: Beyond a phenomenological description of magnetostriction

**DOI:** 10.1038/s41467-018-03389-4

**Published:** 2018-03-07

**Authors:** A. H. Reid, X. Shen, P. Maldonado, T. Chase, E. Jal, P. W. Granitzka, K. Carva, R. K. Li, J. Li, L. Wu, T. Vecchione, T. Liu, Z. Chen, D. J. Higley, N. Hartmann, R. Coffee, J. Wu, G. L. Dakowski, W. F. Schlotter, H. Ohldag, Y. K. Takahashi, V. Mehta, O. Hellwig, A. Fry, Y. Zhu, J. Cao, E. E. Fullerton, J. Stöhr, P. M. Oppeneer, X. J. Wang, H. A. Dürr

**Affiliations:** 1 Stanford Institute for Materials and Energy Sciences, SLAC National Accelerator Laboratory, 2575 Sand Hill Road, Menlo Park, CA 94025 USA; 2 Linac Coherent Light Source, SLAC National Accelerator Laboratory, 2575 Sand Hill Road, Menlo Park, CA 94025 USA; 3 Accelerator Division, SLAC National Accelerator Laboratory, 2575 Sand Hill Road, Menlo Park, CA 94025 USA; 4 Department of Physics and Astronomy, Uppsala University, P.O. Box 516, S-75120 Uppsala, Sweden; 5 Department of Applied Physics, Stanford University, Stanford, CA 94305 USA; 6 CNRS, Laboratoire de Chimie Physique – Matière et Rayonnement, Sorbonne Universités, UPMC Univ. Paris 06, 75005 Paris, France; 7 Van der Waals-Zeeman Institute, University of Amsterdam, 1018XE Amsterdam, The Netherlands; 8 Faculty of Mathematics and Physics, Department of Condensed Matter Physics, Charles University, Ke Karlovu 5, CZ-12116 Prague 2, Czech Republic; 9 Brookhaven National Laboratory, Upton, NY 1193 USA; 10 Department of Physics, Stanford University, Stanford, CA 94305 USA; 11 Stanford Synchrotron Radiation Laboratory, SLAC National Accelerator Laboratory, 2575 Sand Hill Road, Menlo Park, CA 94025 USA; 12 Magnetic Materials Unit, National Institute for Materials Science, Tsukuba, 305-0047 Japan; 13 San Jose Research Center, HGST a Western Digital company, 3403 Yerba Buena Road, San Jose, CA 95135 USA; 14 Thomas J. Watson Research Center, 1101 Kitchawan Road, Yorktown Heights, NY 10598 USA; 15 Institute of Physics, Technische Universität Chemnitz, Reichenhainer Straße 70, D-09107 Chemnitz, Germany; 16 Institute of Ion Beam Physics and Materials Research, Helmholtz–Zentrum Dresden–Rossendorf, 01328 Dresden, Germany; 17 Department of Physics and National High Magnetic Field Laboratory, Florida State University, Tallahassee, FL 32310 USA; 18 Center for Memory and Recording Research, UC San Diego, 9500 Gilman Drive, La Jolla, CA 92093-0401 USA

Correction to: *Nature Communications* 10.1038/s41467-017-02730-7, published online 26 January 2018

The original version of this Article omitted the following from the Acknowledgements:

“The technical support from SLAC Accelerator Directorate, Technology Innovation Directorate, LCLS laser division and Test Facility Division is gratefully acknowledged. We thank S.P. Weathersby, R.K. Jobe, D. McCormick, A. Mitra, S. Carron and J. Corbett for their invaluable help and technical assistance. Research at SLAC was supported through the SIMES Institute which like the LCLS and SSRL user facilities is funded by the Office of Basic Energy Sciences of the U.S. Department of Energy under Contract No. DE-AC02-76SF00515. The UED work was performed at SLAC MeV-UED, which is supported in part by the DOE BES SUF Division Accelerator & Detector R&D program, the LCLS Facility, and SLAC under contract Nos. DE-AC02-05-CH11231 and DE-AC02-76SF00515. Use of the Linac Coherent Light Source (LCLS), SLAC National Accelerator Laboratory, is supported by the U.S. Department of Energy, Office of Science, Office of Basic Energy Sciences under Contract No. DE-AC02-76SF00515.”

and

“Work at BNL was supported by DOE BES Materials Science and Engineering Division under Contract No: DE-AC02-98CH10886. J.C. would like to acknowledge the support from National Science Foundation Grant No. 1207252. E.E.F. would like to acknowledge support from the U.S. Department of Energy (DOE), Office of Basic Energy Sciences (BES) under Award No. DE-SC0003678.”

This has been corrected in both the PDF and HTML versions of the Article.

