# Supplementary material for: Beyond a phenomenological description of magnetostriction
Source: Nat Commun. 2018 Jan 26;9:388. doi: 10.1038/s41467-017-02730-7 (PMC5786062; doi:10.1038/s41467-017-02730-7)
Supplement: Supplementary file 1 — Supplementary Information [file 41467_2017_2730_MOESM1_ESM.pdf]

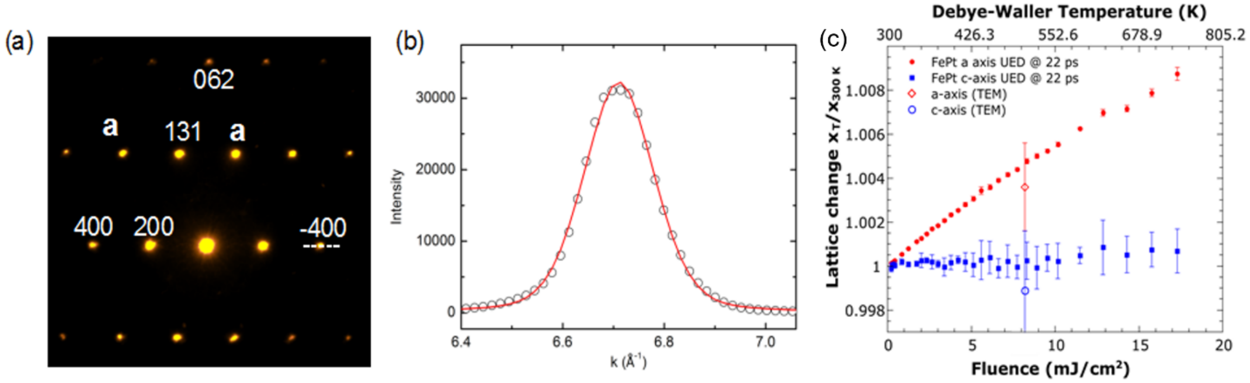

Supplementary Figure 1 (a) Typical electron diffraction pattern in the $(01\bar{3})$ direction of a single FePt nanoparticle. (b) Intensity profile of the (-400) Bragg peak marked by the white line in (a). The reciprocal lattice positions of Bragg peaks are determined by fitting the intensity profile of various diffraction peaks with a combined Gaussian-Lorentz function. (c) Extracted change of *a*- and *c*-axis lattice spacings for warning to 500 K together with UED data as a function of laser fluence (and associated temperature) measured at a pump probe delay of 22 ps (s.d error bars).

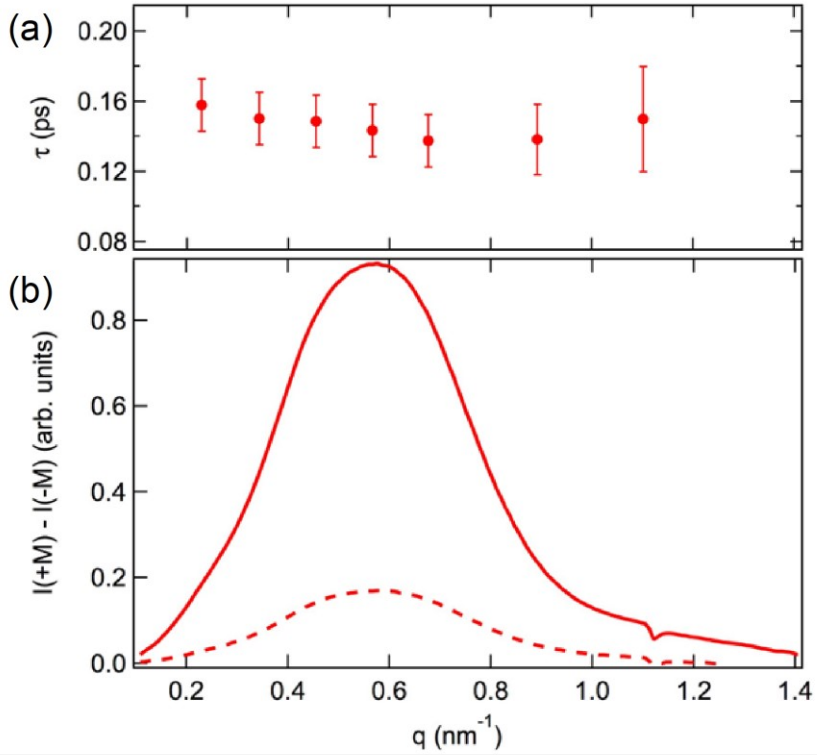

Supplementary Figure 2 (a) demagnetization time constants, τ, obtained from time delay traces taken at the indicated wavevectors, *q* with s.d. error bars. (b) radial diffraction intensity for circularly polarized x-rays with the photon energy tuned into the 2p – 3d core–valence resonance of Fe. The x-ray magnetic dichroism is shown, i.e. the difference in scattering intensity between opposite FePt sample magnetizations. Dashed and solid lines represent data taken with 800 nm pump arriving before and after the x-ray probe pulses, respectively.

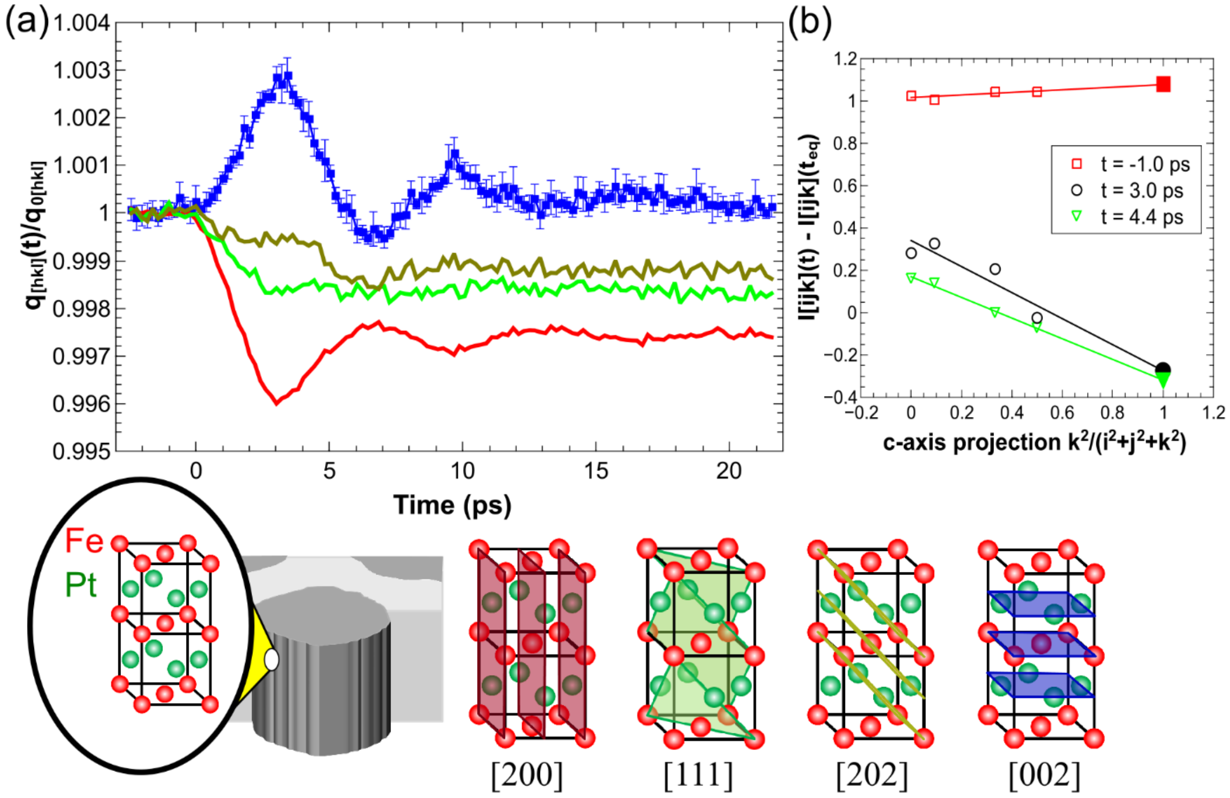

Supplementary Figure 3 (a) Ultrafast electron diffraction time delay traces for the indicated Bragg peak positions with the respective lattice planes indicated schematically in the crystal structures below (s.d error bars). (b) Extrapolation of the measured Bragg reflections by their c-axis (out-of-plane) projection (solid symbols) to the c-axis lattice behavior (open symbols) shown in (a) as blue line and symbols**.**

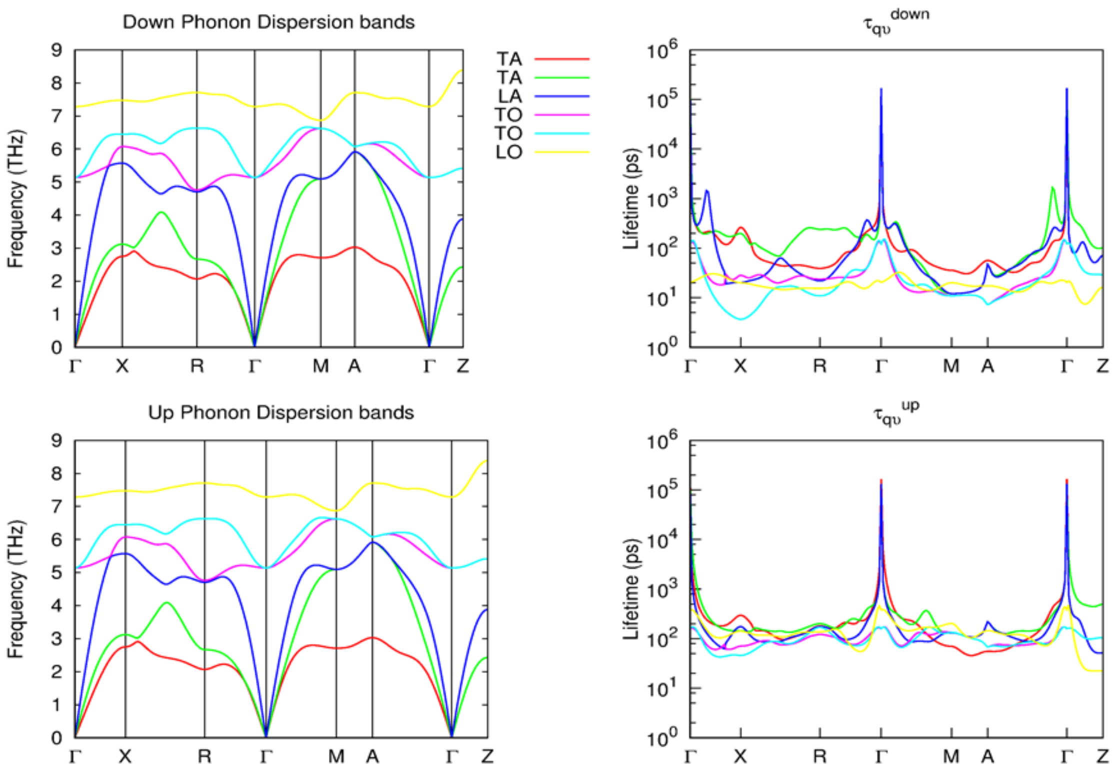

Supplementary Figure 4 Left panels: phonon dispersions calculated for FePt in the $L1_0$ structure for spin-down and spin-up electrons, respectively. Right panels: the corresponding mode-dependent phonon lifetimes due to the electron-phonon coupling for spin-up and spin-down electrons. Different colors indicate different phonon branches. The labels given are those for the high-symmetry points in the tetragonal Brillouin zone.

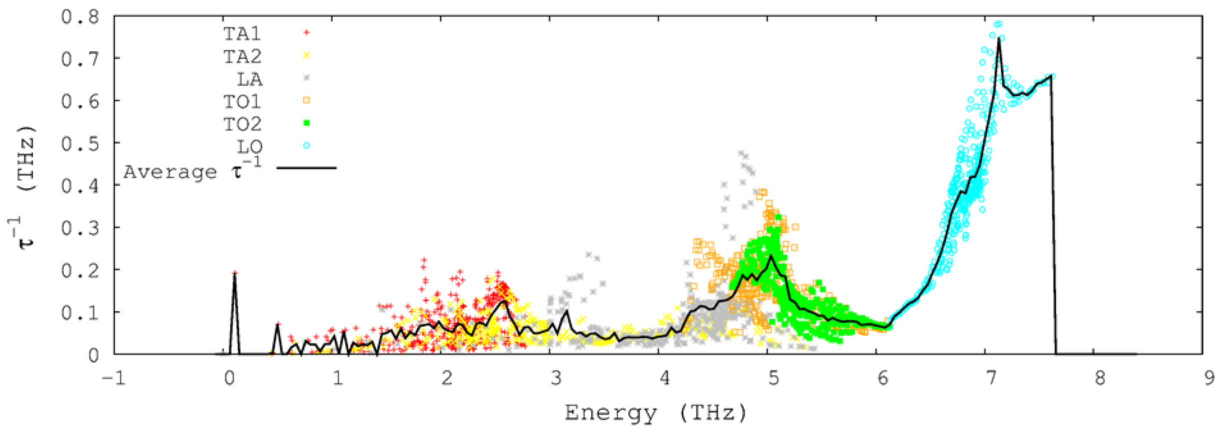

Supplementary Figure 5 Calculated variation of the mode-dependent inverse phonon-phonon lifetime for FePt as function of the phonon frequency. The different phonon branches are indicated with different colors. The average inverse phonon lifetime is depicted by a black line.
